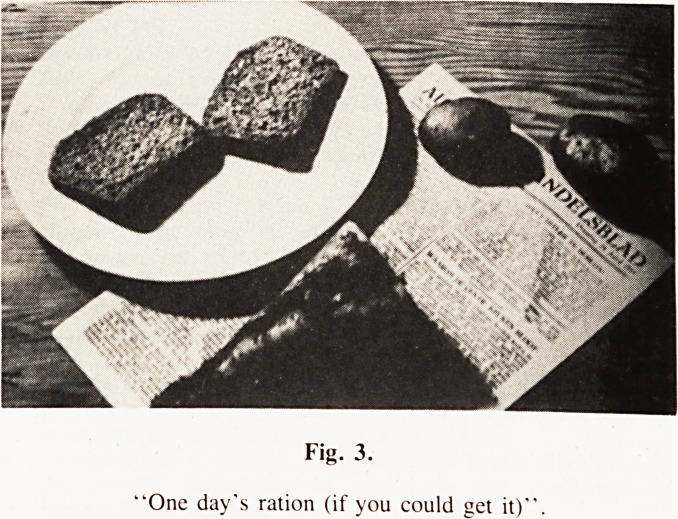# The Mobile Hospitals of the British Red Cross and the Order of St. John and Their Role in the Rotterdam Typhoid Epidemic 1945

**Published:** 1992-08

**Authors:** Lumsden Walker


### The Mobile Hospitals of the British Red Cross and the Order of St. John and their role in the Rotterdam Typhoid Epidemic 1945

**Lumsden Walker**

### THE HOSPITALS AND THEIR PURPOSE

During the planning for the invasion of Mainland Europe in 1944, (D-Day), one consideration was the possibility of there being large numbers of Civilian casualties, which together with the possibility of severe damage to the local Hospitals, might lead to the Military hospitals being unable to cope. The idea therefore arose of creating small 50 bed mobile Hospitals, to care for Civilian wounded or sick, and which with their staff of Medical Officer, Nurses, Driver/Orderlies and Cook, could move wherever needed. They would be run by the Red Cross and St. John organisations, but the staff to be subject to Military control, and hold 'equivalent' military rank.

In the event, there was as all know, very fierce fighting in North West France but relatively few Civilian casualties, despite the extensive destruction of towns and villages, particularly in Normandy. The need to supply the advancing allied armies meant that there was no available space for the planned Mobile Hospitals in the first months following D-Day. By the first week of September, Allied troops were already in Brussels, but not until late Autumn did the first of the mobile hospitals cross into Belgium, to be almost captured in the German Ardennes offensive. The rest of the five Mobile hospitals followed in the succeeding two months. It might at that point have been queried what their purpose could be, but the situation developing in Holland, and later in Germany meant that they were to be more than fully occupied. This paper however will be concerned only with the work of Hospitals 4 and 5 in Rotterdam.

### THE SITUATION IN HOLLAND

The failure of the Airborne attack on Arnhem in September of 1944 meant that the Arnhem Bridge over the Rhine remained in German hands (the 'Bridge Too Far'). The subsequent Allied advance into Germany left almost all of northern Holland, with all the major Cities, including Amsterdam and Rotterdam, under German occupation, and this continued until the very end of the European war in May of 1945. Grave shortages of Food, Fuel and Transport arose and increased rapidly. By the Autumn of 1944 the situation of the Civilian Population was already becoming desperate. Mortality and morbidity, particularly from Starvation, Tuberculosis and other Infectious Illnesses increased dramatically. The daily individual ration had fallen from 1500 Calories in the Summer of 1944 to 500 to 600 Calories by February 1945. Insulin, Sulphonamides and indeed most medicaments were no longer available. Transport was non existent. Those who still had pedal cycles had no tyres.

**Fig. 1.**

"October 1944 to May 1945: The large Cities remain Occupied".

**Fig. 2.**

"The desperate search for firewood: Verboten van de Deutsche Weermacht".

As Winter approached Electricity (October 9) and Gas supplies (October 19) previously severely limited, were cut off. Doctors Surgeries and Hospitals had limited supplies, but none after February of '45. Trees, wood from abandoned bombed buildings, even rail sleepers were taken and cut up by people desperate for some warmth. Part-cooked Tulip and Dahlia bulbs became food. The Death Rate rose by 232% for Males, by 75% for Females and by over 140% for Children, by comparison with the first half of 1944.

**Fig. 3.**

"One day's ration (if you could get it)".

In 1945, in the City of Rotterdam there were over 3,000 cases of Diptheria, a massive increase of Tuberculosis, and inevitably the hospitals were in addition filled by the victims of prolonged starvation.

### THE TYPHOID EPIDEMIC

The village of Spijkenisse, then some 10 miles outside Rotterdam, had a water supply from pumps and shallow wells. Thanks to military defensive flooding, the cess-pits of the houses began to drain into the local canal, and then into the wells. The billeting of a German soldier, returned from the East front, and a Typhoid carrier, had the inevitable result. There was no fuel. Water could not be boiled, nor Milk (if available) pasteurised, nor food properly cooked. There was an inevitable rapid spread of infection.

| Month | Notifications |
|---|---|
| April | 3 |
| May | 73 |
| June | 226 |
| July | 72 |
| August | 73 |

By August the epidemic was being controlled. Food and fuel supplies were returning to normal, as were the Health Department's effective measures, (including sealing the pumps, and importing pure water from the city's intact piped supply).

Initially however the hospitals, already full and understaffed by people themselves ill from cold, hunger and years of fear under occupation, were unable to cope.

This was clearly the situation for which the mobile hospitals were envisaged, but on a much larger scale. Hospital No. 4 was sent in, and almost immediately had to call for the help of No. 5. The Dutch authorities gave the use of the former Port Quarantine hospital, evacuated only 2 or 3 days before by the German Navy, and very much damaged. Then came the task of admitting the patients with Typhoid from their homes as quickly as possible, to prevent further epidemic spread. The task was to turn two 50 bed emergency hospitals into an acute Isolation hospital. Within two weeks they had achieved 200+ beds. Domestic and Nursing staff were urgently recruited locally, together with three Dutch and two Swiss Doctors, including a Bacteriologist (Dutch) and a Technician plus a mobile laboratory (British).

Hard work was needed to get the badly destroyed kitchens working again before the patients were able to take solid food, but in the event proved successful.

### TREATMENT

Sulphathiozole was the main medication available. Two children with a complicating Bronchopneumonia were successfully given the only Penicillin available. It is generally accepted however that outcome depends mainly on skilled Nursing. It says much for the Nursing Staff, many with only Auxilliary Nurse training, and of two Nationalities, that the death rate was, in these adverse conditions remarkably low.

Of the total of 558 recognised cases of Typhoid in Rotterdam and its conurbation, 264 (almost half) were treated in the Mobile Hospitals. Of these 264 there were 15 deaths (a mortality rate of 5.6%). The mortality rate overall, for all cases was 7.5%. After the first two months the Mobile hospitals were taking the less severe cases as their stay was to be limited to just over 4 months, but this was not so initially. This is a low mortality rate, despite preceding cold and starvation. It is perhaps of interest that Murchison in 1873 wrote "The rate of mortality was not greater amongst the destitute than in the better classes". Perhaps this **was** a factor. It is also of interest that of 146 Children under the age of 14 years, only 4 died, and the others made a good recovery.

As patients improved, and during convalescence, were checked for Carrier status, the Hospitals were given the use of a Hospital Barge moored on the river as a recovery unit. This relieved pressure on the other beds, and improved their well-being and mood during recovery.

### IN CONCLUSION

By enabling rapid removal of patients from their homes (almost half of all notified cases) the Mobile hospitals, must have played a vital part in controlling the outbreak. By late Summer, the local hospitals were once again able to accept all admissions and to them the few remaining patients were transferred. The Red Cross and St. John hospitals and their augmented staff moved on to the next emergency situation in camps for "Displaced Persons" in Germany.

### REFERENCES

BANNING C. and LOHR H. (1947). Occupied Holland. *BMJ.* **1.** p539.

BANNING C. (1945). Gezondheidsbestand in Nederland: in Nederlands Tijdschrift voor Geneskunde. **89.** p311.

VAN DER ZEE (1982). The Hunger Winter (Occupied Holland). Published: Norman & Hobhouse Ltd. London.

## Figures and Tables

**Fig. 1. f1:**
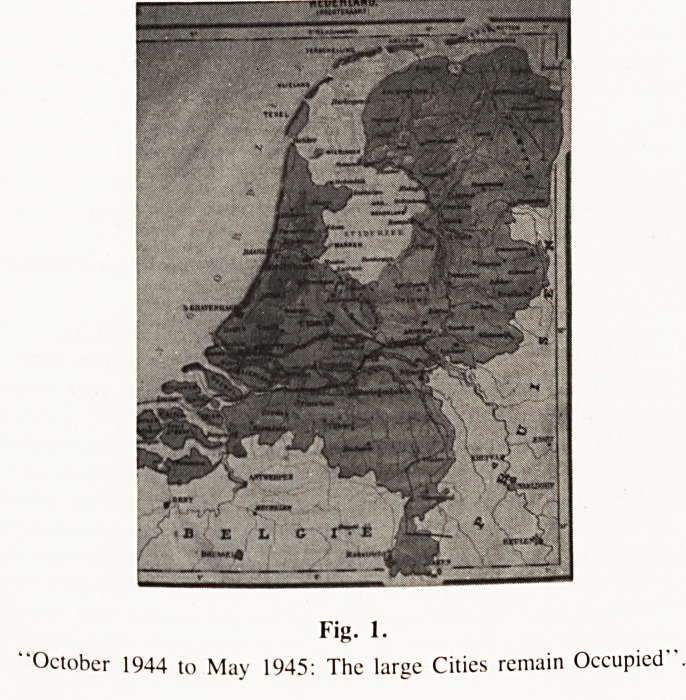


**Fig. 2. f2:**
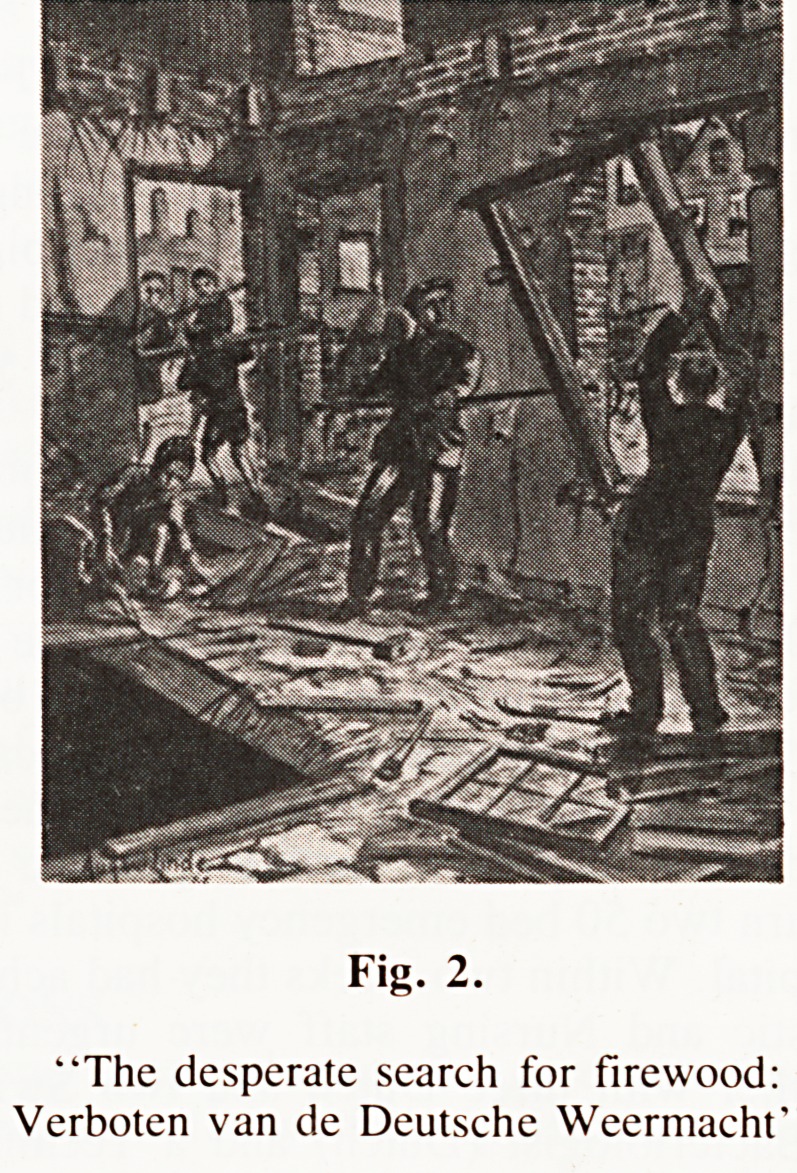


**Fig. 3. f3:**